# The effects of n-6 polyunsaturated fatty acids on the expression of nm-23 in human cancer cells.

**DOI:** 10.1038/bjc.1998.120

**Published:** 1998-03

**Authors:** W. G. Jiang, S. Hiscox, R. P. Bryce, D. F. Horrobin, R. E. Mansel

**Affiliations:** University Department of Surgery, University of Wales College of Medicine, Heath Park, Cardiff, UK.

## Abstract

**Images:**


British Journal of Cancer (1998) 77(5), 731–738
© 1998 Cancer Research Campaign

### The effects of *n*-6 polyunsaturated fatty acids on the expression of *nm-23* in human cancer cells

**WG Jiang[1], S Hiscox[1], RP Bryce[2], DF Horrobin[2] and RE Mansel[1]**

Metastasis Research Group, [1]University Department of Surgery, University of Wales College of Medicine, Heath Park, Cardiff, UK; [2]Scotia Pharmaceuticals, Stirling, Scotland, UK

**Summary** This study examined the effect of *n*-6 polyunsaturated fatty acids (PUFAs) on the expression of *nm-23*, a metastasis-suppressor gene, in two highly invasive human cancer cell lines, HT115 and MDA MB 231. A range of *n*-6 and *n*-3 PUFAs were tested. We report that while linoleic acid and arachidonic acid reduced the expression of *nm-23*-H1, gamma linolenic acid (GLA) and its soluble lithium salt markedly increased the expression of the molecules. The stimulation of the expression of *nm-23* by GLA was seen at both protein and mRNA levels. Up-regulation of *nm-23* was also associated with a reduction of the in vitro invasiveness of these cells. It is concluded that gamma linolenic acid (GLA) enhances the expression of *nm-23*. This contributes to the inhibition of the in vitro invasion of tumour cells.

**Keywords:** *nm-23*; gamma linolenic acid; invasion; metastasis; polyunsaturated fatty acid

*nm-23* is a known metastasis-suppressor gene (Steeg et al, 1988; Bevelacqua et al, 1989; Rosengard et al, 1989; for reviews see Steeg et al, 1993; MacDonald et al, 1995; Rosa et al, 1995). Three human *nm-23* genes have been identified, designated *nm-23*-H1, *nm-23*-H2 and DR-*nm-23* (Venturelli et al, 1995). H1 and H2 encode nucleoside diphosphate kinase (NDPK) A and NDPK B polypeptides respectively. Both in vivo and in vitro, the *nm-23* gene and expression of its protein product correlate with non-metastatic behaviour of cancer cells. In vitro, the motility and the invasiveness of tumour cells inversely correlate with the level of *nm-23*; transfection of highly invasive cells with *nm-23* cDNA results in a reduction or complete inhibition of invasiveness (Leone et al, 1991, 1993; Kantor et al, 1993). Conversely, experimental deletion of the *nm-23* gene results in a highly invasive cell phenotype. Recent studies by Hsu et al (1995) have implicated *nm-23* in the regulation of signal transduction pathways used by motility factors.

In both animal and clinical studies, *nm-23* levels have been found to be decreased in tumour cells and tissues, and this reduction has been shown to be closely related to disease stage, presence of metastases and prognosis. Reduction of *nm-23* levels has been observed in patients with colorectal cancer (Yamaguchi et al, 1993; Campo et al, 1994; Royds et al, 1994), breast cancer (Hennessey et al, 1991; Tokunaga et al, 1993; Noguchi et al, 1994; Simpson et al, 1994), liver cancer (Iizuka et al, 1995), melanoma (Xerri et al, 1994), oesophageal cancer, bladder cancer (Fujii et al, 1995), ovarian cancer (Mandai et al, 1995; Viel et al, 1995) and several other tumour types (Rosa et al, 1995). Interestingly, however, this relationship is not seen in thyroid cancer (Holm et al, 1995). In the early stages of colorectal cancer, it seems that there may be an overexpression of both *nm-23*-H1 and H2, but at advanced stages there is a marked reduction of *nm-23*-H1 protein (Martinez et al, 1995). Colorectal cancer may also be associated with mutations of *nm-23* (Wang et al, 1993). Missense mutations and loss of heterozygosity of *nm-23* have also been reported in both ovarian serous carcinoma (Mandai et al, 1995) and primary breast cancer (Cropp et al, 1994). In ovarian tumours, reduction of *nm-23* is related to the lymphatic dissemination of tumour cells (Viel et al, 1995), and in breast cancer it has been suggested that impairment of *nm-23* is correlated with lymph node involvement (Noguchi et al, 1994).

In a number of studies, some essential fatty acids have been shown to be selectively toxic to tumour cells: among these are gamma linolenic acid, a member of the *n*-6 series and eicosapentaenoic acid, a member of the *n*-3 series of essential fatty acids (for a review see Horrobin, 1990). However another study failed to show a clear pattern of the selectivity among normal and tumorigenic cells (Maehle et al, 1995). These fatty acids have been tested on a range of cancer cell types, including lung, breast, prostate, pancreatic cancer and hepatoma cells (Begin et al, 1986, 1988; Botha et al, 1989; Newman, 1990; Rose et al, 1991; Tiwari et al, 1991; Hayashi et al, 1992; Takeda et al, 1992, 1993). Furthermore, the inhibition of tumour cell growth by certain cytokines is dependent on the presence of polyunsaturated fatty acids (PUFAs) (Newman, 1990). Lipid peroxides have been shown to be important factors responsible for *n*-6 fatty acid-induced cytotoxicity (Horrobin, 1990).

We have previously investigated the role of essential fatty acids (EFAs) in the invasion and metastatic behaviour of cancer cells and have shown that certain EFAs, including gamma linolenic acid (GLA), produce a marked inhibition of the motility/invasiveness and metastatic properties of cancer cells. These are effects that may arise in part by the up-regulation of cell surface E-cadherin and other related molecules (Jiang et al, 1995*a* and *b*). However, GLA also inhibits the motility and in vitro invasiveness of tumour cells that have been shown to be E-cadherin negative (e.g. HT115 human colon cancer and MDA MB 231 human breast cancer cells) (Jiang et al, 1995 *a* and *b*) and so other mechanisms must also be operative.

*Received 22 April 1997*
*Revised 1 July 1997*
*Accepted 3 July 1997*

*Correspondence to:* WG Jiang, Metastasis Research Group, University Department of Surgery, University of Wales College of Medicine, Cardiff CF4 4XN, UK

These observations led us to look for other metastatic parameters that might contribute to an explanation of the anti-invasion effects exerted by these fatty acids. We report here that GLA, at non-toxic concentrations, up-regulates the expression of *nm-23-*H1 in HT115 and MDA MB 231 cells. This regulation appears to be at a transcriptional level, as both Western and Northern blotting revealed increased protein and mRNA expression in response to GLA treatment. This change in *nm-23* levels correlates with a reduction in the in vitro invasiveness of these cells.

### MATERIALS AND METHODS

A human colon cancer cell line, HT115, and a human breast cancer cell line, MDA-MB-231, were obtained from the European Collection of Animal Cell Culture (ECACC, Porton Down, Salisbury, UK) and the ATCC (American Type Cell Collection, Rockville, Maryland, USA) respectively. Cells were routinely cultured with Dulbecco's modified Eagle medium (DMEM) supplemented with 10% fetal calf serum (FCS).

Matrigel (reconstituted basement membrane) was purchased from Collaborative Research Products (Bedford, Massachusetts, USA). A transwell plate equipped with a porous insert (pore size 8.0 μm) was from Becton Dickinson Labware (Oxford, UK) and used for the in vitro invasion study. A mouse anti-human *nm-23*-H1 monoclonal antibody was from Santa-Cruz Biotechnology (Autogen Bioclear UK, Devizes, Wilts, UK). Peroxidase-conjugated rabbit anti-mouse IgG for both immunohistochemical studies and Western blotting was from Amersham International (Little Chalfont, Buckinghamshire, UK). Recombinant human hepatocyte growth factor/scatter factor (HGF/SF) was a generous gift from Dr T Nakamura, Osaka, Japan. The cells were passaged three to five times before assays were undertaken. Hoescht 33258, gamma linolenic acid (GLA), linoleic acid (LA), arachidonic acid (AA) and eicosapentaenoic acid (EPA) were from Sigma-Aldrich (Poole, Dorset, UK). A water-soluble lithium salt of GLA was from Callanish (Isle of Lewis, Scotland). All the fatty acids were dissolved in ethanol and stored in liquid nitrogen before use. Fatty acids were diluted in culture medium with 10% FCS, with the final concentration of ethanol being less than 0.01% (Jiang et al, 1995*a*). A cDNA probe for human *nm-23*-H1 was used for Northern blotting studies and was obtained from ATCC (American Type Culture Collection, Rockville, MD, USA). All other materials were purchased from Sigma-Aldrich unless otherwise stated.

### Cell invasion assay

This was based on the methods of Albini et al (1987) and Parish et al (1992). Transwell chambers (Costar, Cambridge, MA, USA) equipped with a 6.5-mm-diameter polycarbonate membrane (pore size 8 μm) were precoated with a solubilized tissue basement membrane (Matrigel; 50 μg per membrane). After gel rehydration, 50 000 cells were added to each membrane with or without treatment. Hepatocyte growth factor/scatter factor (20 ng ml$^{-1}$) was used in the lower chamber to induce invasion. After a 72-h culture, the non-invasive cells were removed with a cotton swab and the cells that had migrated through the membrane and stuck to the lower surface were fixed and stained with crystal violet. After extraction with 10% acetic acid, the absorbance was measured at 540 nm with a Titertek multiscanner.

### SDS-PAGE and Western blotting

To test the effects of fatty acids on *nm-23* expression, a range of five fatty acids were used at 50 μM (a concentration that we have previously shown to be non-toxic to these cells; Jiang et al, 1995*a* and *b*) for 24 h. Cells were also treated with specific fatty acid at a range of concentrations over various periods. After treatment with fatty acids, cells were pelleted and lysed in HCMF buffer containing 1% Triton, 0.1% sodium dodecyl sulphate (SDS), 2 mM calcium chloride, 100 μg ml$^{-1}$ phenylmethysulphonyl fluoride (PMSF), 1 μg ml$^{-1}$ leupeptin and 1 μg ml$^{-1}$ aprotinin for 30 min. They were then boiled at 100°C for 5 min before clarification at 13 000 *g* for 10 min.

**Figure 1** The effect of fatty acids on the expression of *nm-23*-H1 in HT115 cells (Western blotting). Fatty acids were used at 50 μM and cultured with cells for 24 h. Selective up-regulation of *nm-23* was seen with GLA and LiGLA. AA, arachidonic acid; LiGLA, GLA lithium salt; LA, linoleic acid; EPA, eicosapentaenoic acid. Top, nm23 protein probed with anti-nm23 antibody; bottom, nm23 protein band volume obtained from densitometry

**Figure 2** Effect of fatty acids on the expression of *nm-23*-H1 in MDA MB 231 cells (Western blotting). Experimental conditions were as in Figure 1

© Cancer Research Campaign 1998

**Figure 3** Immunocytochemical detection of *nm-23* in HT115 (**A** and **B**) and MDA MB 231 (**C** and **D**) cells. **A** and **C**, control; **B** and **D**, cells treated with GLA at 50 μM for 24 h. There was an increased intracellular staining of *nm-23* after GLA treatment

**Figure 4** Concentration-dependent stimulation of *nm-23* by GLA in HT115 cells by Western blotting. The treatment was for 24 h. The increased expression was seen at concentrations over 10 μM. Top, nm23 protein probed with anti-nm23 antibody; bottom, nm23 protein band volume obtained from densitometry

Protein concentrations were measured using fluorescamine and quantified by using a multifluoroscanner (Denly, Sussex, UK). Equal amounts of protein from each cell sample (30 μg per lane) (controls and treated) were added on to a 12% polyacrylamide gel. After electrophoresis, proteins were blotted on to nitrocellulose sheets and blocked in 10% skimmed milk for 60 min before probing with the *nm-23* antibody (1:1500) and a peroxidase-conjugated secondary antibody (1:2000). A low-molecular-weight marker mixture (SDS-7, Sigma) was used to determine the protein size. Protein bands were visualized with an enhanced chemiluminescence (ECL) system (Amersham International, UK). Protein band densities were measured with a laser densitometer and band volumes were analysed with the Molecular Analyst software (Bio-Rad Laboratories, Hemel Hempstead, Hertfordshire, UK).

### Northern and slot blotting

Cells in 75-cm$^2$ flasks were treated with fatty acids for 4, 12, 24, 48 and 72 h or over a range of fatty acid concentrations (1.0–150 μM) for 24 h. Some cells were treated with different fatty acids at fixed concentration for 24 h for comparison between these different fatty acids. Total cellular RNA was extracted as previously described (Chomczynski and Sacchi, 1987). For Northern analysis, 10 μg of total RNA were resolved on a 0.8% denaturing agarose gel and transferred to a nylon membrane. A cDNA probe for human *nm-23*-H1 was used for subsequent hybridization overnight at 45°C in the presence of formamide. Membranes were washed under stringent conditions and then exposed to radiographic film. Slot blots were also performed on the cellular RNA using a slot blotter (Whatman, Maidstone, Kent, UK). All the blots were subsequently re-probed with a human β-actin cDNA to correct for loading errors. mRNA band densities of both *nm-23* and actin were similarly determined using laser densitometry, and *nm-23* levels are shown here as the ratio of *nm-23* in treated samples vs control. The formula used to calculate these ratios is: (nm23 signal in treated cells/nm23 signal in control)/(actin signal in treated cells/actin signal in control).

© Cancer Research Campaign 1998 

**Figure 5** Concentration-dependent stimulation of *nm-23* by GLA in MDA MB 231 cells. The experimental conditions were as in Figure 4

**Figure 6** Time-related effects of GLA on the expression of *nm-23* in MDA MB 231 cells by Western blotting. GLA was used at 50 µM. An increased protein level was seen 12 h after GLA treatment. Top, nm23 protein probed with anti-nm23 antibody; bottom, nm23 protein band volume obtained from densitometry

**Figure 7** Effects of fatty acids on *nm-23* mRNA levels in HT115 cells determined by Northern blotting. The experimental conditions were as in Figure 1. Top, *nm-23* probed mRNA; bottom, changes in nm23 mRNA level in fatty acid treated vs control with loading errors corrected by β-actin signals. Shown in the figure are ratios calculated as (nm23 signal in treated cells/nm23 signal in control)/(actin signal in treated cells/actin signal in control). Increased *nm-23* mRNA was seen after GLA treatment of the cell

**Figure 8** Effects of GLA at different concentrations on *nm-23* mRNA levels in HT115 cells determined by Northern blotting. Cells were treated with GLA for 24 h. Top, *nm-23* probed mRNA; bottom, changes in nm23 mRNA level. Shown are ratios of the changes with loading errors corrected by β-actin signals (as in Figure 7). GLA at concentrations higher than 10 µM showed a stimulatory effect

### Immunohistochemical study

Cells were fixed with 4% formaldehyde and permeabilized with 0.01% Triton X100 for 5 min before blocking with 10% milk for 60 min. Cells were then incubated with *nm-23* monoclonal antibody for 60 min and extensively washed. After this, peroxidase-conjugated secondary antibody was added and the colour developed using diaminobenzidine (DAB, 180 µg $ml^{-1}$ containing 0.03% hydrogen peroxide). Slides were mounted with slide mountant Sterilyte (BDH) and photographed.

### Cell growth monitoring

In all the studies, cellular DNA content from both controls and fatty acid-treated samples was determined using Hoescht 33258 (final concentration 1.0 µg $ml^{-1}$; Jiang et al, 1995*b*) to assess fatty acid cytotoxicity. Briefly, at the end of each experiment, the DNA from control and fatty acid-treated cells was extracted with 0.1% SDS for 60 min. To the cell lysate was added the fluorescent indicator Hoescht 33258 (Sigma) (final concentration 1.0 µg $ml^{-1}$). The fluorescence was monitored by a Wellfluo multiscanner (Denly, Sussex, UK) at exitation 356 nm and emission 458 nm.

© Cancer Research Campaign 1998

**Figure 9** Effect of GLA on the in vitro invasion of MDA MB 231 cells. **A**, Cells cultured with medium as control; **B**, cells treated with GLA at 50 μM for 72 h; **C**, cells treated with HGF/SF (20 ng ml$^{-1}$ final concentration); **D**, cells treated with HGF/SF plus GLA at 50 μM; **E**, invasion as determined by crystal violet assay (details given in Materials and methods). Shown are invasion indices obtained from the spectrophotometer. MDA MB 231 cells were invasive even without any stimulation (**A**). This invasion was enhanced when HGF/SF was included (**C**). Inclusion of GLA in the culture system reduced both spontaneous and HGF/SF-stimulated invasiveness (**B** and **D**). **E**, GLA significantly reduced the in vitro invasion, particularly that induced by HGF/SF. *$P < 0.05$ vs cell with medium alone; **$P < 0.05$ vs cells with HGF/SF alone

### RESULTS

### Expression of *nm-23*-H1 in tumour cells

To confirm the expression of *nm-23* in the cells used, Northern and Western blotting, together with immunocytochemistry, were carried out. Western blotting showed a single band of protein at approximately 18 kDa recognized by anti-*nm-23*-H1 antibody. Northern blotting revealed an approximate 1-kb transcript in both cell types. Immunocytochemistry confirmed that the staining of *nm-23*-H1 was located in the cytosolic region of both cells.

### Effects of fatty acids on *nm-23* expression

We then tested the effect of a range of fatty acids on *nm-23* protein expression. Total cellular protein was prepared from each sample for Western blotting. Of all the fatty acids tested, gamma linolenic acid and its lithium salt showed an up-regulatory effect on the *nm-23* protein level in both cell types (Figure 1, HT115 cells; Figure 2, MDA MB 231 cells). In contrast, linoleic acid (LA) and arachidonic acid showed slight inhibitory effects. EPA had little effect.

This GLA-induced increase in nm23 expression was also confirmed by immunocytochemistry (Figure 3).

© Cancer Research Campaign 1998

### Concentration-dependent effects of GLA on *nm-23* expression

GLA was then tested over a range of concentrations (1–150 μM). The effect of GLA on the expression of *nm-23* was seen from 10 μM (Figure 4, HT115; Figure 5, MDA MB 231 cells), with a maximal effect occurring at approximately 50 μM. A concentration of 50 μM was chosen for a time course study ranging from 4 to 72 h. The maximum increase of protein was seen 12 h after initial exposure (Figure 6).

### Regulation of *nm-23* mRNA by fatty acids

Northern blotting studies showed that *nm-23* mRNA was selectively increased in response to GLA and LiGLA (Figure 7). This was also seen in a concentration-dependent manner (Figure 8). The time course and concentration response were further determined and quantified with a slot blot, which revealed that the *nm-23* levels were seen to increase 4 h after treatment but returned to near starting levels after 48 h. This time course indicated that the regulation of *nm-23* expression by fatty acids was occurring at the transcription level.

### The role of GLA on tumour cell in vitro invasion

Using an in vitro invasion model, we then tested the effect of GLA on cell invasion of an extracellular matrix, Matrigel. At non-toxic concentrations, GLA inhibited both spontaneous and hepatocyte growth factor/scatter factor (HGF/SF)-induced cell invasion (Figure 9). Both cell types showed a similar response to GLA.

### Measurement of fatty acid cytotoxicity

In order to determine whether the observed effects arising after fatty acid treatment were attributed to cytotoxicity, cell growth was monitored using a Hoescht 33258 DNA assay. At the fatty acid concentrations used in this study, the cellular DNA content was not affected.

### DISCUSSION

In this paper, we have shown that the polyunsaturated fatty acid gamma linolenic acid selectively up-regulates the expression of the metastasis-suppressor gene *nm-23* in cells that are highly invasive. This effect was not due to fatty acid cytotoxicity.

GLA and other fatty acids have been shown to be toxic to a range of human tumour cells, an effect that is thought to result in part from the fatty acid-induced production of superoxide within the cell (Horrobin, 1990). At lower concentrations, GLA and related fatty acids may be involved in the regulation of the motility and invasive behaviour of cancer cells, and we have recently shown that GLA is capable of inhibiting both spontaneous and motogen-stimulated cell motility and invasion in vitro (Jiang et al, 1995*a*, 1996, 1997*a*). Previous studies have suggested that this is likely to be partly the result of up-regulation of the cell-surface adhesion molecule E-cadherin and desmosomal cadherin (desmoglein), which are recognized metastasis-suppressor molecules (Jiang et al, 1995*a* and *b*, 1997*b*). However, such inhibition also occurred in cells that were shown to be E-cadherin negative by Western blotting (e.g. HT115 and MDA MB 231 cells), suggesting that GLA exerts its invasion/motility inhibitory effects via an E-cadherin-independent mechanism. The data reported here clearly show that GLA regulates the expression of *nm-23*, and this correlates with a decreased invasive potential of these tumour cells. We propose, therefore, that *nm-23* regulation may represent another pathway by which GLA can inhibit cellular motility and invasion.

This study also shows that arachidonic acid (AA) reduced the expression of *nm-23*. It has previously been shown that one of the arachidonic acid metabolites, prostaglandin E2 (PGE2), also suppresses *nm-23* expression (Parhar et al, 1995). This indicates that gamma linolenic acid exerts different effects compared with some of its other metabolites (i.e. AA and PGE2).

Diverse effects on cell behaviour are also seen with other arachinonic acid metabolites. Prostacyclin has been shown to exert an anti-metastatic effect, possibly by regulation of tumour-related aggregation (Schneider et al, 1994). In contrast, 12(s)-HETE has been reported to be pro-invasive and pro-metastatic by influencing cell motility and invasion (Honn et al, 1992, 1994). Furthermore, prostacyclin antagonizes the effect of 12(S)-HETE (Schneider et al, 1994). While these effects by prostacyclin and 12(S)-HETE are mediated by their receptors, the mechanisms of the effects of GLA and other EFAs are less clear as no receptors have been identified for these fatty acids.

Work by Howlett et al (1994) has shown that *nm-23* exerts a novel function in breast cancer cells by inducing matrix deposition and therefore basement membrane formation and growth arrest. Although our study does not provide data on matrix deposition, we have shown a strong correlation between the increased expression of *nm-23* and reduction of basement membrane invasion by these cells, an effect consistent with the work of Howlett et al (1994).

The serine phosphorylation of *nm-23* correlates with reduced metastatic activity of tumour cells (Macdonald et al, 1993) and, in future studies, it will be useful to determine whether GLA is able to promote such phosphorylation.

The data presented here may have clinical relevance. Patients receiving GLA as a component of a treatment regimen may benefit from the effect on *nm-23* seen in vitro. In some but not all studies performed in both humans and animals bearing breast or liver tumours, *n*-6 PUFAs have been shown to have beneficial effects (Karmali et al, 1985; Ramchurren et al, 1985; McIllmurray and Turkie, 1987; Van der Merwe et al, 1988, 1990; Pritchard and Mansel, 1990). In recent studies, intravenous delivery of gamma linolenic acid to patients with advanced pancreatic cancer showed improvement in patients survival (Fearon et al, 1996), as did the local application of GLA into the brain in patients with malignant glioma (Das et al, 1995).

In summary, this study shows that polyunsaturated fatty acids are able to regulate the expression of *nm-23* in human cancer cells. Such regulation is likely to be at the transcription level. Up-regulation of metastasis-suppressor genes, such as *nm-23*, may prove useful in the treatment of cancer patients.

### ACKNOWLEDGEMENT

The authors would like to thank th‹• Welsh Scheme for the Development of Health and Social Research for supporting this work.

### REFERENCES

Albini A, Iwamoto Y, Kleinman HK, Martin GR, Aaronson SA, Kozlowski JM and McEwan RN (1987) A rapid *in vitro* assay for quantitating the invasive potential of tumor cells. *Cancer Res* **47**: 3239–3245

© Cancer Research Campaign 1998

Begin ME, Ells G, Das UN and Horrobin DF (1986) Differential killing of human carcinoma cells supplemented with n-3 and n-6 polyunsaturated fatty acids. *J Natl Cancer Inst* **77**: 1053–1062

Begin ME, Ells G, Das UN and Horrobin DF (1988) Polyunsaturated fatty acids induced cytotoxicity against tumor cells and its relationship to lipid peroxidation. *J Natl Cancer Inst* **80**: 188–194

Bevilacqua G, Sobel ME, Liotta LA and Steeg PS (1989) Association of low *nm-23* rna levels in human primary infiltrating ductal breast carcinomas with lymph-node involvement and other histopathological indicators of high metastatic potential. *Cancer Res* **49**: 5185–5190

Botha JH, Robinson KM, Ramchurren N and Norman RJ (1989) The role of prostaglandins in the inhibition of cultured carcinoma cell growth produced by gammalinolenic acid. *Prostaglandin Leukotr Essent Fatty Acid* **35**: 119–123

Campo E, Miquel R, Jares P, Bosch F, Juan M, Leone A, Vives J, Cardesa A and Yague J (1994) Prognostic-significance of the loss of heterozygosity of *nm-23*-H1 and p53 genes in human colorectal carcinomas. *Cancer* **73**: 2913–2921

Chomczynski P and Sacchi N (1987) A single step method of RNA isolation by acid guanidimium thiosulphate–phenol–chloroform extraction. *Anal Biochem* **162**: 156–159

Cropp CS, Lidereau R, Leone A, Liscia D, Cappa APM, Campbell G, Barker E, Ledoussal V, Steeg PS and Callahan R (1994) NME1 protein expression and loss of heterozygosity mutations in primary human breast-tumors. *J Natl Cancer Inst* **86**: 1167–1169

Das UN, Prasad VSK and Reddy DR (1995) Local application of gamma-linolenic acid in the treatment of human gliomas. *Cancer Lett* **94**: 147–155

Fearon KCH, Falconer JS, Ross JA, Carter DC, Hunter JO, Reynolds PD and Tuffnell Q (1996) An open-label phase i/ii dose-escalation study of the treatment of pancreatic-cancer using lithium gammalinolenate. *Anticancer Res* **16**: 867–874

Fujii K, Yasui W, Shimamoto F, Yokozaki H, Nakayama H, Kajiyama G and Tahara E (1995) Immunohistochemical analysis of *nm-23* gene-product in human gallbladder carcinomas. *Virch Arch* **426**: 355–359

Hayashi Y, Fukushima S, Kishimoto S, Kawaguchi T, Numata M, Isoda Y, Hirano J and Nakano M (1992) Anticancer effects of free polyunsaturated fatty acids in an oily lymphographic agent following intrahepatic arterial administration to a rabbit bearing VX-2 tumor. *Cancer Res* **52**: 400–405

Hennessy C, Henry JA, May FEB, Westley BR, Angus B and Lennard TWJ (1991) Expression of the antimetastatic gene *nm-23* in human breast cancer – an association with good prognosis. *J Natl Cancer Inst* **83**: 281–285

Holm R, Hoie J, Kaalhus O and Nesland JM (1995) Immunohistochemical detection of *nm-23*/NDP kinase and cathepsin-D in medullary carcinomas of the thyroid-gland. *Virch Arch* **427**: 289–294

Honn KV, Nelson KK, Renaud C, Bazaz R, Diglio CA and Timar J (1992) Fatty acid modulation of tumor cell adhesion to microvessel endothelium and experimental metastasis. *Prostaglandins* **44**: 413–429

Honn KV, Tang DG, Gao X, Butovich IA, Liu B, Timar J and Hagmann W (1994) 12-lipoxygenases and 12(S)-HETE: role in cancer metastasis. *Cancer Metastasis Rev* **13**: 365–396

Horrobin DF (1990) Essential fatty acids, lipid peroxidation, and cancer. In *Omega-6 Essential Fatty Acids,* Horrobin DF. (ed.), pp. 351–378. Wiley-Liss: New York.

Howlett AR, Petersen OW, Steeg PS and Bissell MJ (1994) A novel function for the *nm-23*-H1 gene – overexpression in human breast-carcinoma cells leads to the formation of basement-membrane and growth arrest. *J Natl Cancer Inst* **86**: 1838–1844

Hsu S, Huang F, Ossowski L and Friedman E (1995) Colon-carcinoma cells with inactive *nm-23* show increased motility and response to motility factors. *Carcinogenesis* **16**: 2259–2262

Iizuka N, Oka M, Noma T, Nakazawa A, Hirose K and Suzuki T (1995) *Nm-23*-H1 and *nm-23*-H2 messenger-RNA abundance in human hepatocellular-carcinoma. *Cancer Res* **55**: 652–657

Jiang WG, Hiscox S, Hallett MB, Horrobin DF, Scott C and Puntis MCA (1995*a*) Inhibition of invasion and motility of human colon cancer cells by gamma linolenic acid. *Br J Cancer* **71**: 744–752

Jiang WG, Hiscox S, Hallett MB, Horrobin DF, Mansel RE and Puntis MCA (1995*b*) Regulation of the expression of E-cadherin on human cancer cells by gamma linolenic acid. *Cancer Res* **55**: 5043–5048

Jiang WG, Hiscox S, Hallett MB, Bryce R, Horrobin DF, Mansel RE and Puntis MCA (1996) Inhibition of membrane ruffling and ezrin translocation by gamma linolenic acid. *Int J Oncol* **9**: 279–284

Jiang WG, Bryce RP and Mansel RE (1997*a*) Gamma linolenic acid regulates gap junction communications in endothelial cells and their interaction with tumour cells. *Prostaglandin Leukotr Essent Fatty Acid* **56**: 307–316

Jiang WG, Singhrao SK, Hiscox S, Hallett MB, Bryce PB, Horrobin DF, Puntis MCA and Mansel RE (1997*b*) Regulation of desmosomal cell adhesion in human tumour cells by polyunsaturated fatty acids. *Clin Exp Metastasis* **15**: 593–602

Kantor JD, McCormick B, Steeg PS and Zetter BR (1993) Inhibition of cell motility after *nm-23* transfection of human and murine tumor-cells. *Cancer Res* **53**: 1971–1973

Karmali RA, Marsh J and Fuchs C (1985) Effects of dietary enrichment with gamma linolenic acid upon growth of the R3230AC mammary adenocarcinoma. *J Nutr Growth Cancer* **2**: 41–51

Leone A, Flatow U, King CR, Sandeen MA, Margulies IMK, Liotta LA and Steeg PS (1991) Reduced tumor-incidence, metastatic potential, and cytokine responsiveness of *nm-23*-transfected melanoma-cells. *Cell* **65**: 25–35

Leone A, Flatow U, Vanhoutte K and Steeg PS (1993) Transfection of human *nm-23*-H1 into the human MDA MB435 breast-carcinoma cell-line-effects on tumor metastatic potential, colonization and enzymatic-activity. *Oncogene* **8**: 2325–2333

McIllmurray MB and Turkie W (1987) Controlled trial of gamma linolenic acid in Dukes's colorectal cancer. *Br Med J* **294**: 1260

Macdonald NJ, Delarosa A, Benedict MA, Freije JMP, Krutsch H and Steeg PS (1993) A serine phosphorylation of *nm-23*, and not its nucleoside diphosphate kinase-activity, correlates with suppression of tumor metastatic potential. *J Biol Chem* **268**: 25780–25789

Macdonald NJ, Delarosa A and Steeg PS (1995) The potential roles of *nm-23* in cancer metastasis and cellular-differentiation. *Eur J Cancer* **31A**: 1096–1100

Maehle L, Eilertsen E, Mollerup S, Schonberg S, Krokan HE and Haugen A (1995) Effects of n-3 fatty acids during neoplastic progression and comparison of in vitro and in vivo sensitivity of 2 human tumor cell lines. *Br J Cancer* **71**: 691–696

Mandai M, Konishi I, Komatsu T, Mori T, Arao S, Nomura H, Kanda Y, Hiai H and Fukumoto M (1995) Mutation of the *nm-23* gene, loss of heterozygosity at the *nm-23* locus and k-ras mutation in ovarian carcinoma – correlation with tumor progression and *nm-23* gene expression. *Br J Cancer* **72**: 691–695

Martinez JA, Prevot S, Nordinger B, Nguyen TMA, Lacarriere Y, Munier A, Lascu I, Vaillant JC, Capeau J and Lacombe ML (1995) Overexpression of *nm-23*-H1 and *nm-23*-H2 genes in colorectal carcinomas and loss of *nm-23*-H1 expression in advanced tumor stages. *Gut* **37**: 712–720

Newman MJ (1990) Inhibition of carcinoma and melanoma cell growth by type 1 transforming growth factor beta is dependent on the presence of polyunsaturated fatty acids. *Proc Natl Acad Sci USA* **87**: 5543–5547

Noguchi M, Earashi M, Ohnishi I, Kitagawa H, Fusida S, Miyazaki I and Mizukami Y (1994) Relationship between *nm-23* expression and axillary and internal mammary lymph-node metastases in invasive breast-cancer. *Oncol Rep* **4**: 795–799

Parhar RS, Shi YF, Zou MJ, Farid NR, Ernst P and Alsedairy ST (1995) Effects of cytokine-mediated modulation of *nm-23* expression on the invasion and metastatic behavior of B16f10 melanoma-cells. *Int J Cancer* **60**: 204–210

Parish CR, Jakobsen KB and Coombe DR (1992) A basement membrane permeability assay which correlates with the metastatic potential of tumour cells. *Int J Cancer* **52**: 378–383

Pritchard GA and Mansel RE (1990) The effects of essential fatty acids on the growth of breast cancer and melanoma. In *Omega-6 Essential Fatty Acids* Horrobin DF. (ed.), pp. 379–390. Wiley-Liss: New York

Ramchurren N, Botha JH and Leary WP (1985) An investigation into the effects of gamma linolenic acid on murine sarcoma M52B. *S Afr J Sci* **81**: 331

Rosa ADL, Williams RL and Steeg PS (1995) *Nm-23*/nucleoside diphosphate kinase: toward a structural and biochemical understanding of its biological functions. *Bioessays* **17**: 53–62

Rose DP, Connolly JM and Meschter CL (1991) Effect of dietary fat on human breast cancer growth and lung metastasis in nude mice. *J Natl Cancer Inst* **83**: 1491–1495

Rosengard AM, Krutzsch HC, Shearn A, Biggs JR, Barker E, Margulies IMK, King CR, Liotta LA and Steeg PS (1989) Reduced *nm-23* and protein in tumor-metastasis and aberrant drosophila development. *Nature* **342**: 177–180

Royds JA, Cross SS, Silcocks PB, Scholefield JH, Rees RC and Stephenson TJ (1994) *NM-23* antimetastatic gene-product expression in colorectal carcinoma. *J Pathol* **172**: 261–266

Shneider MR, Tang DG, Schirner M and Honn KV (1994) Prostacyclin and its analogues: antimetastatic effects and mechanisms of action. *Cancer Metastasis Rev* **13**: 349–364

Simpson JF, Omalley F, Dupont WD and Page DL (1994) Heterogeneous expression of *nm-23* gene-product in noninvasive breast-carcinoma. *Cancer* **73**: 2352–2358

Steeg PS, Bevilacqua G, Pozzatti R, Liotta LA and Sobel ME (1988) Altered expression of *nm-23*, a gene associated with low tumor metastatic potential,

© Cancer Research Campaign 1998

during adenovirus-2 ela inhibition of experimental metastasis. *Cancer Res* **48**: 6550–6554

Steeg PS, Delarosa A, Flatow U, Macdonald NJ, Benedict M and Leone A (1993) *NM-23* and breast-cancer metastasis. *Breast Cancer Res Treat* **25**: 175–187

Takeda S, Horrobin DF, Manku M, Sim PG, Ells G and Simmons V (1992) Lipid peroxidation in human breast cancer cells in response to gamma-linolenic acid and iron. *Anticancer Res* **12**: 329–333

Takeda S, Sim PG, Horrobin DF, Sanford T, Chisholm KA and Simmons V (1993) Mechanism of lipid peroxidation in cancer cells in response to gamma-linolenic acid (GLA) analyzed by Gc-Ms(I): conjugated dienes with peroxyl (or hydroperoxyl) groups and cell-killing effects. *Anticancer Res* **13**: 193–199

Tiwari RK, Mukhopadhyay B, Telang NT and Osborne MP (1991) Modulation of gene expression by selected fatty acids in human breast cancer cells. *Anticancer Res* **11**: 1383–1388

Tokunaga Y, Urano T, Furukawa K, Kondo H, Kanematsu T and Shiku H (1993) Reduced expression of *nm-23*-H1, but not of *nm-23*-H2, is concordant with the frequency of lymph-node metastasis of human breast-cancer. *Int J Cancer* **55**: 66–71

Van der Merwe CF, Booyens J and Katzeff IE (1988) Oral gamma linolenic acid in 21 patients with untreatable malignancy. *Br J Clin Pract* **41**: 907–915

Van der Merwe CF, Booyens J, Joubert HF et al (1990) The effect of gamma-linolenic acid, an in vitro cytostatic substance contained in evening primrose oil, on primary liver-cancer – a double-blind placebo controlled trial. *Prostaglandins* **40**: 199–202

Venturelli D, Martinez R, Melotti P, Casella I, Peschle C, Cucco C, Spampinato G, Darzynkiewicz Z, Calabretta B (1995) Overexpression of dr-*nm-23*, a protein encoded by a member of the *nm-23* gene family, inhibits granulocyte differentiation and induces apoptosis in 32DC13 myeloid cells. *Proc Natl Acad Sci USA* **92**: 7435–7439

Viel A, Dallagnese L, Canzonieri V, Sopracordevole F, Capozzi E, Carbone A, Visentin MC and Boiocchi M (1995) Suppressive role of the metastasis-related *nm-23*-H1 gene in human ovarian carcinomas – association of high messenger-RNA expression with lack of lymph-node metastasis. *Cancer Res* **55**: 2645–2650

Wang LM, Patel U, Ghosh L, Chen HC and Banerjee S (1993) Mutation in the *nm-23* gene is associated with metastasis in colorectal-cancer. *Cancer Res* **53**: 717–720

Xerri L, Grob JJ, Battyani Z, Gouvernet J, Hassoun J and Bonerandi JJ (1994) *Nm-23* expression in metastasis of malignant-melanoma is a predictive prognostic parameter correlated with survival. *Br J Cancer* **70**: 1224–1228

Yamaguchi A, Urano T, Fushida S, Furukawa K, Nishimura G, Onemura Y, Miyazaki I, Nakagawara G and Shiku H (1993) Inverse association of *nm-23*-H1 expression by colorectal cancer with liver metastasis. *Br J Cancer* **68**: 1020–1024

© Cancer Research Campaign 1998


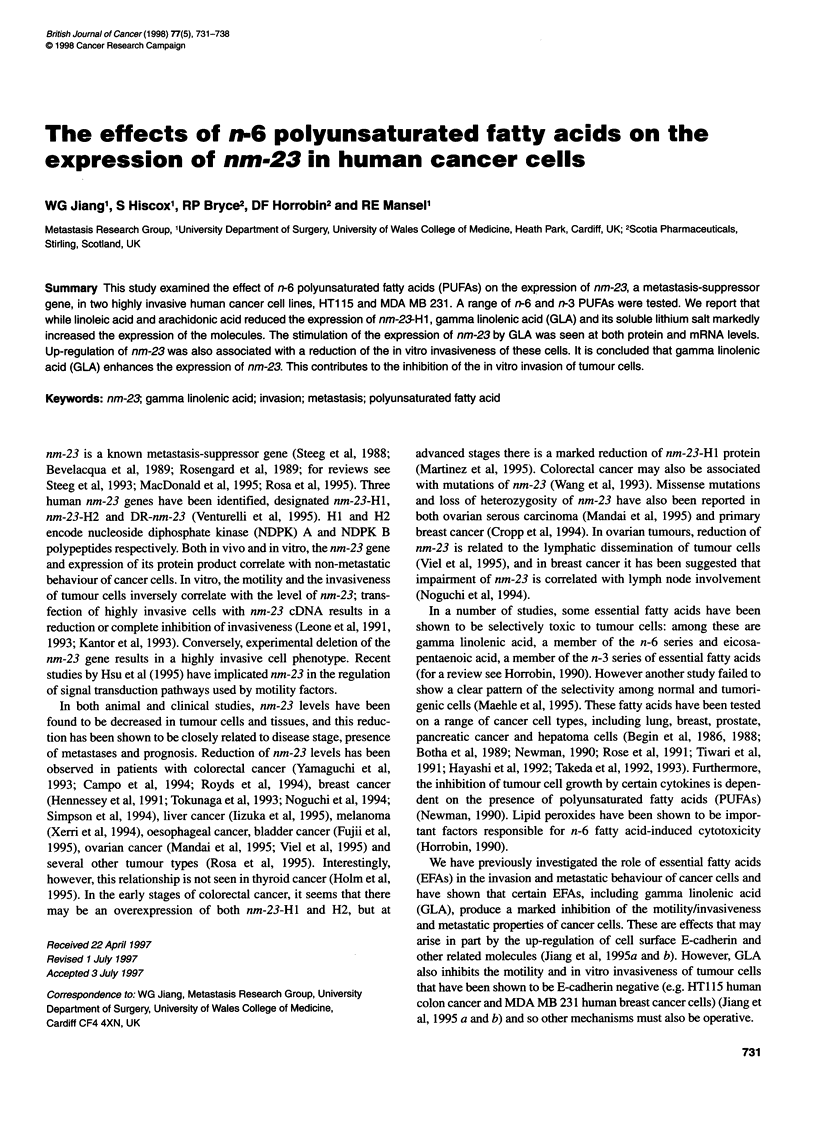

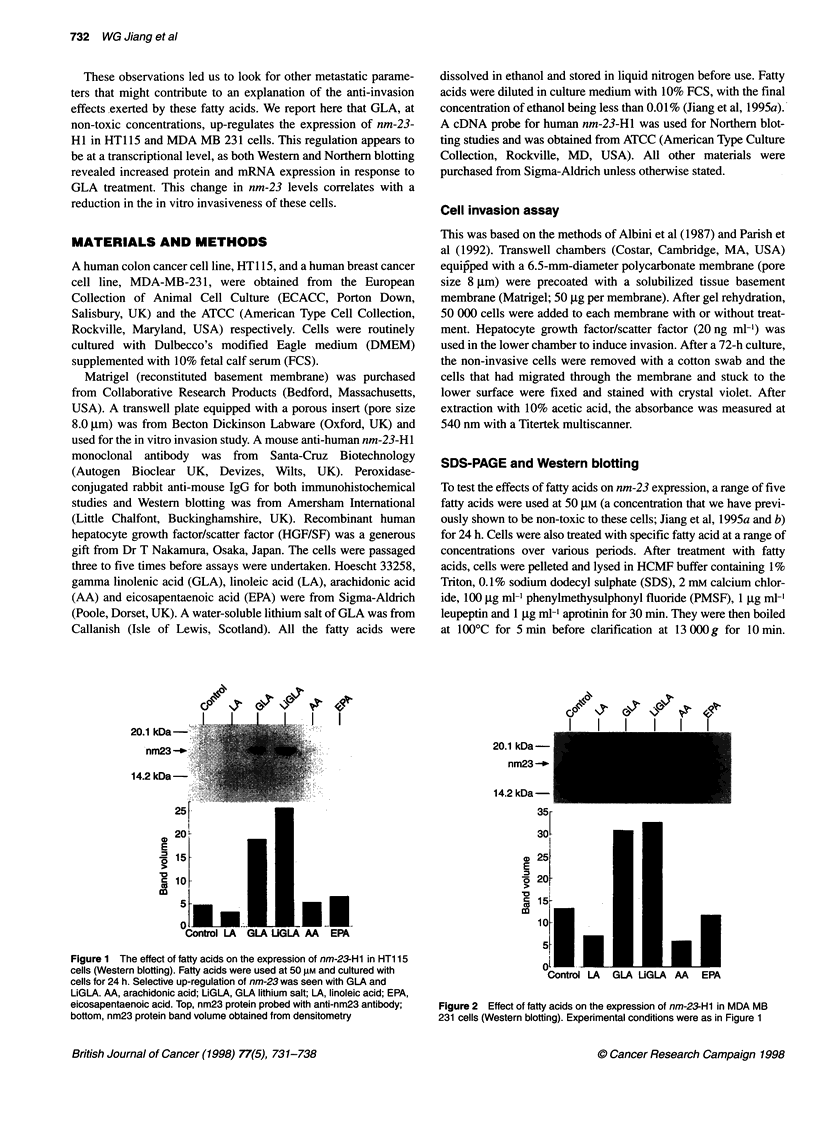

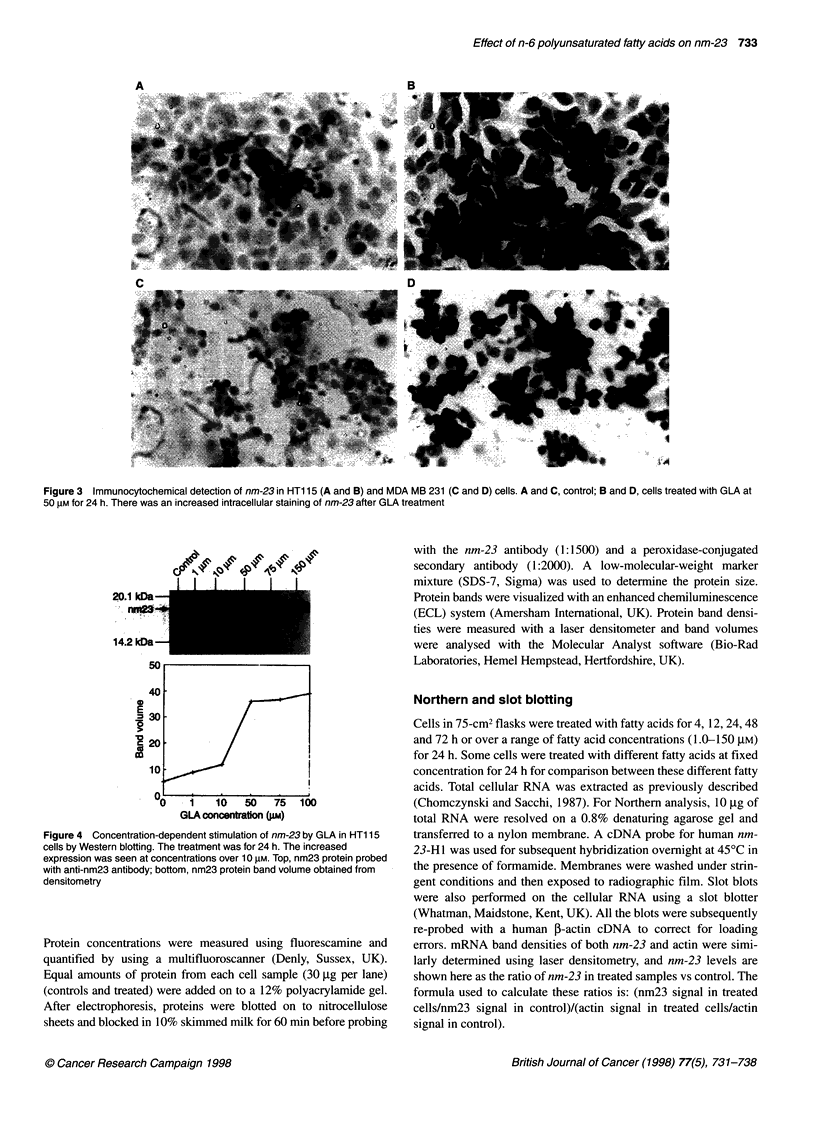

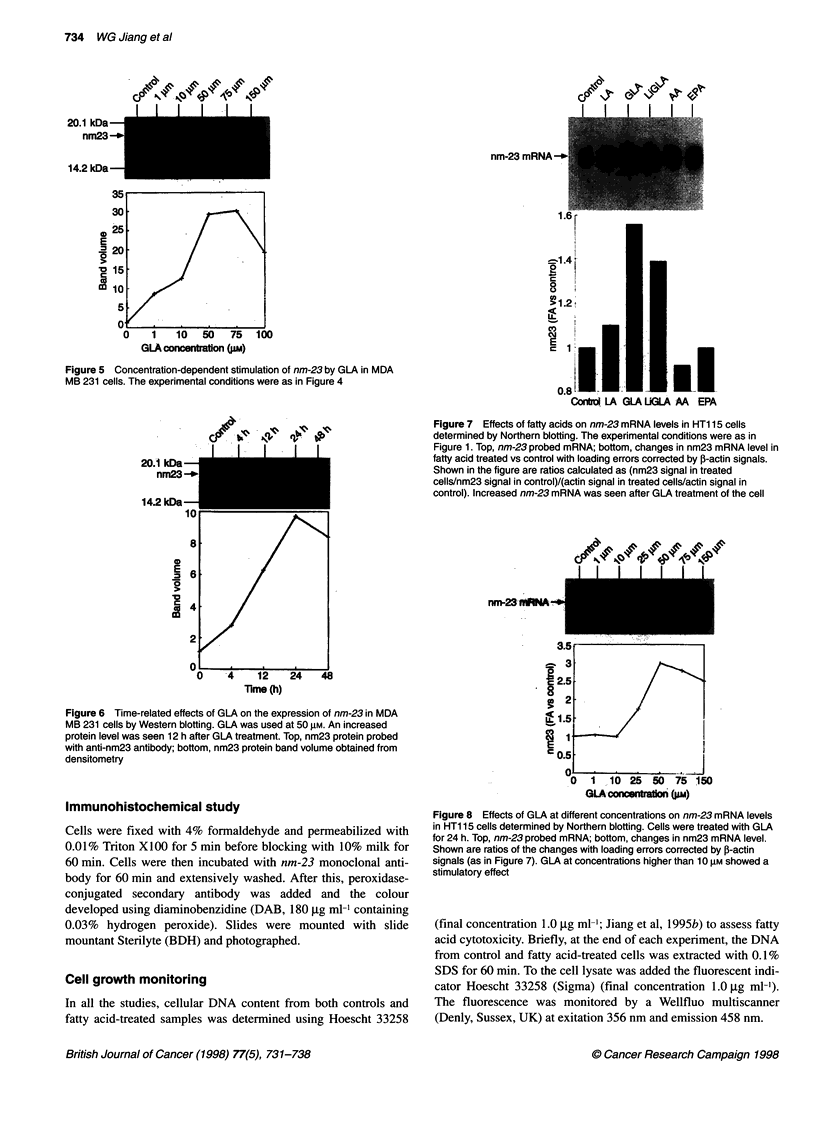

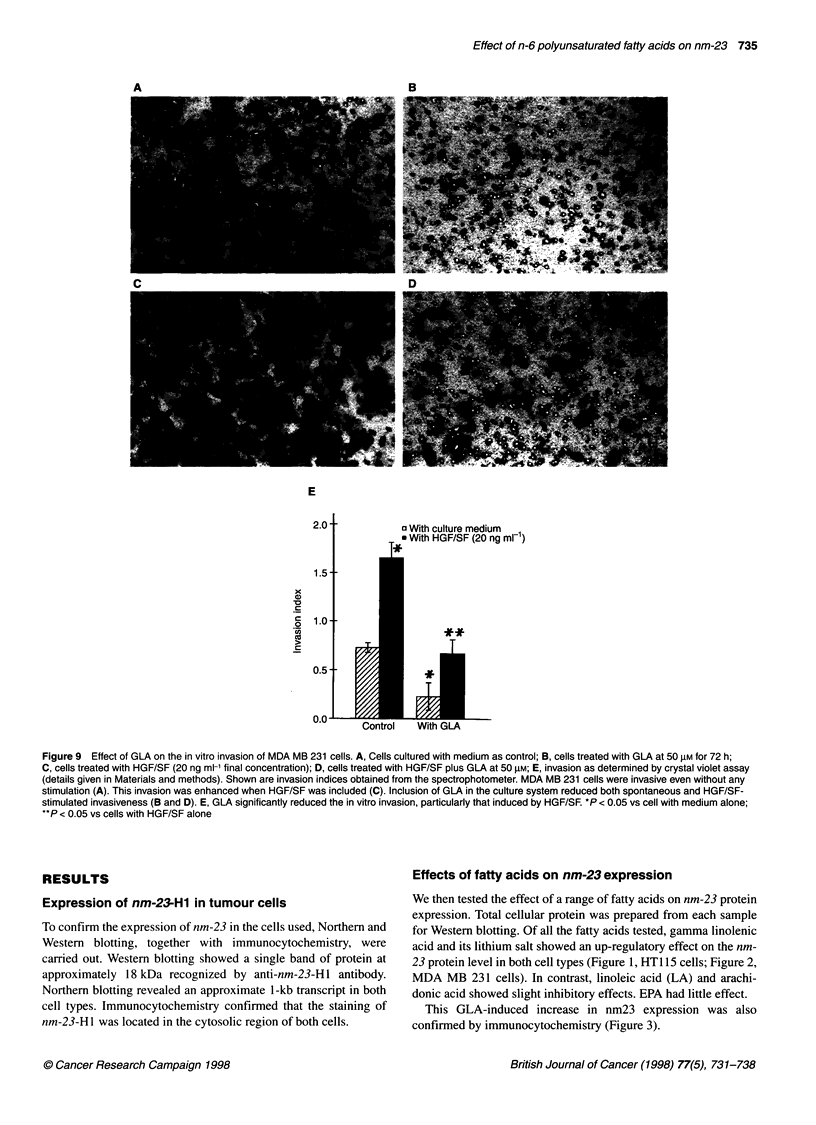

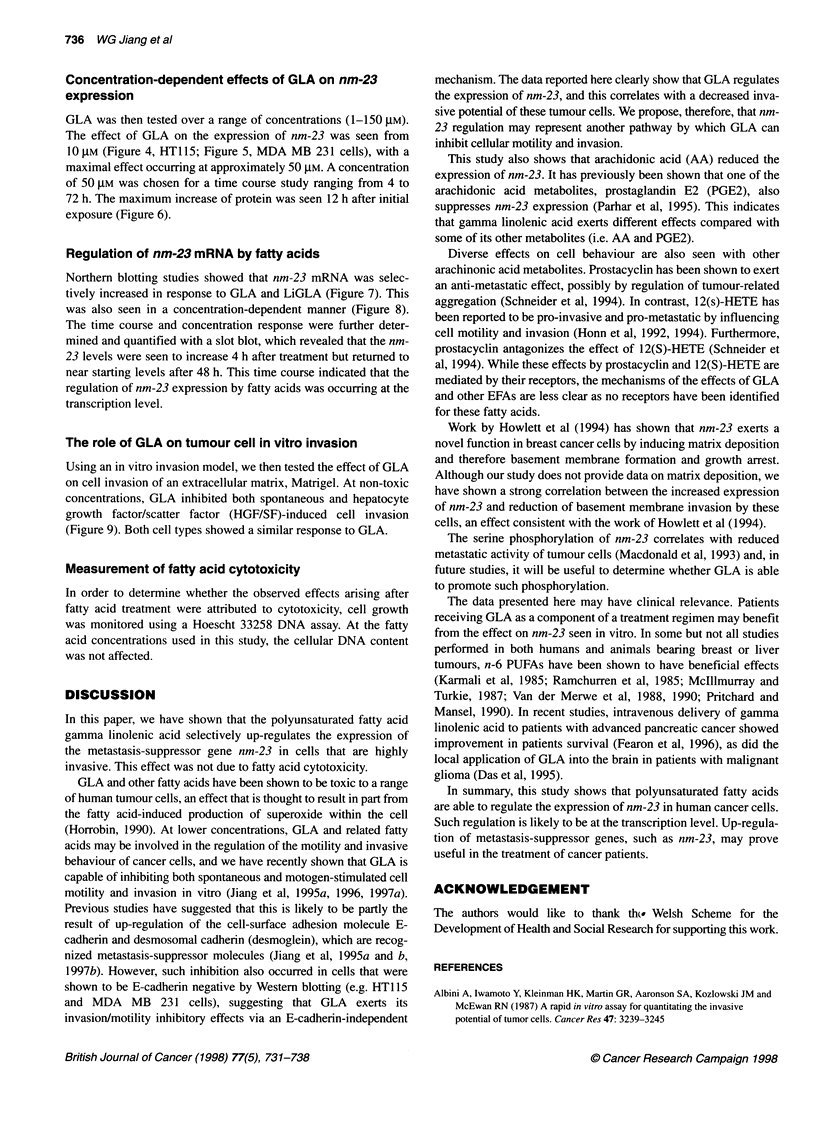

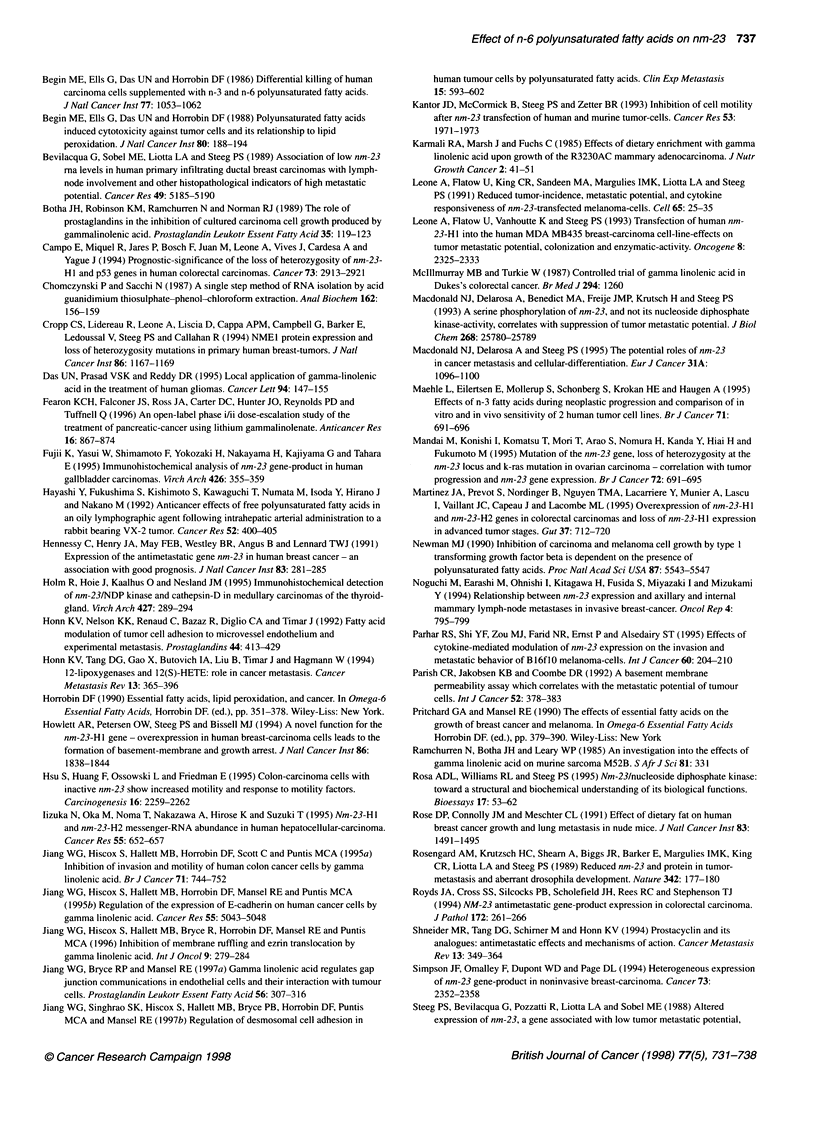

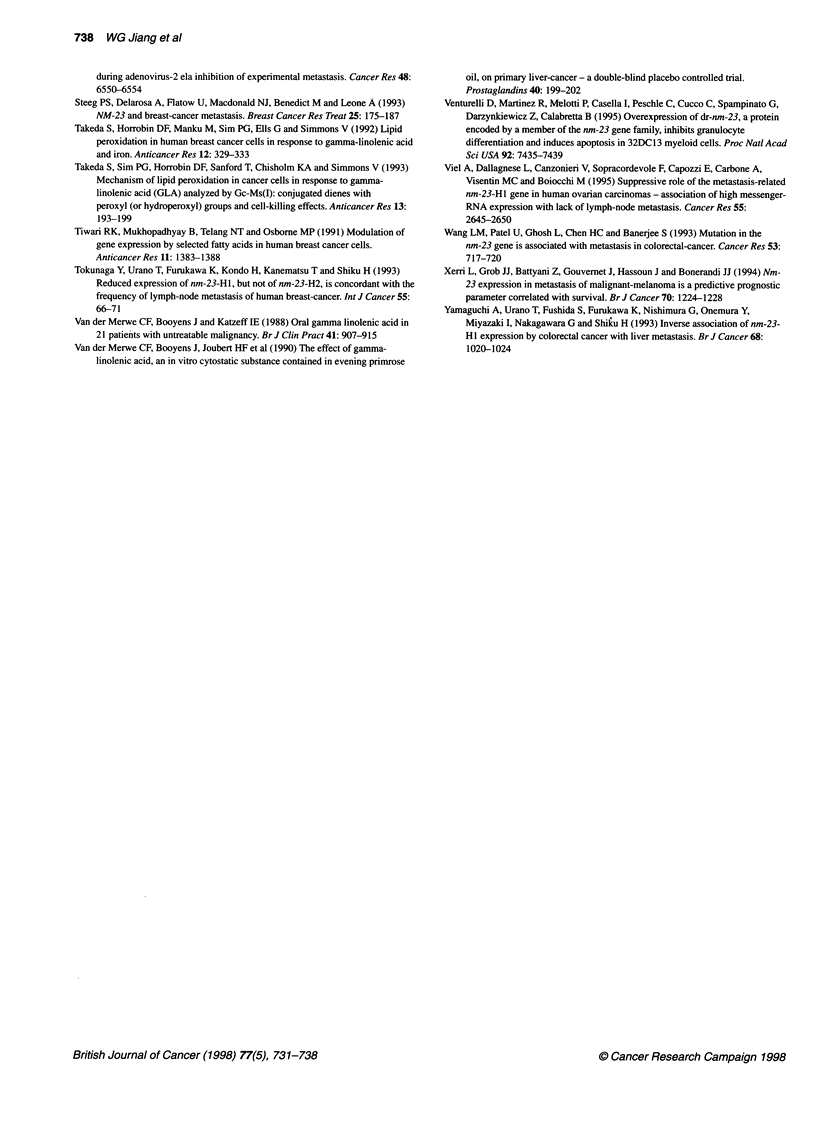


## References

[OCR_00658] Albini A., Iwamoto Y., Kleinman H. K., Martin G. R., Aaronson S. A., Kozlowski J. M., McEwan R. N. (1987). A rapid in vitro assay for quantitating the invasive potential of tumor cells.. Cancer Res.

[OCR_00679] Bevilacqua G., Sobel M. E., Liotta L. A., Steeg P. S. (1989). Association of low nm23 RNA levels in human primary infiltrating ductal breast carcinomas with lymph node involvement and other histopathological indicators of high metastatic potential.. Cancer Res.

[OCR_00685] Botha J. H., Robinson K. M., Ramchurren N., Norman R. J. (1989). The role of prostaglandins in the inhibition of cultured carcinoma cell growth produced by gamma-linolenic acid.. Prostaglandins Leukot Essent Fatty Acids.

[OCR_00669] Bégin M. E., Ells G., Das U. N., Horrobin D. F. (1986). Differential killing of human carcinoma cells supplemented with n-3 and n-6 polyunsaturated fatty acids.. J Natl Cancer Inst.

[OCR_00674] Bégin M. E., Ells G., Horrobin D. F. (1988). Polyunsaturated fatty acid-induced cytotoxicity against tumor cells and its relationship to lipid peroxidation.. J Natl Cancer Inst.

[OCR_00689] Campo E., Miquel R., Jares P., Bosch F., Juan M., Leone A., Vives J., Cardesa A., Yague J. (1994). Prognostic significance of the loss of heterozygosity of Nm23-H1 and p53 genes in human colorectal carcinomas.. Cancer.

[OCR_00694] Chomczynski P., Sacchi N. (1987). Single-step method of RNA isolation by acid guanidinium thiocyanate-phenol-chloroform extraction.. Anal Biochem.

[OCR_00699] Cropp C. S., Lidereau R., Leone A., Liscia D., Cappa A. P., Campbell G., Barker E., Le Doussal V., Steeg P. S., Callahan R. (1994). NME1 protein expression and loss of heterozygosity mutations in primary human breast tumors.. J Natl Cancer Inst.

[OCR_00705] Das U. N., Prasad V. V., Reddy D. R. (1995). Local application of gamma-linolenic acid in the treatment of human gliomas.. Cancer Lett.

[OCR_00709] Fearon K. C., Falconer J. S., Ross J. A., Carter D. C., Hunter J. O., Reynolds P. D., Tuffnell Q. (1996). An open-label phase I/II dose escalation study of the treatment of pancreatic cancer using lithium gammalinolenate.. Anticancer Res.

[OCR_00716] Fujii K., Yasui W., Shimamoto F., Yokozaki H., Nakayama H., Kajiyama G., Tahara E. (1995). Immunohistochemical analysis of nm23 gene product in human gallbladder carcinomas.. Virchows Arch.

[OCR_00721] Hayashi Y., Fukushima S., Kishimoto S., Kawaguchi T., Numata M., Isoda Y., Hirano J., Nakano M. (1992). Anticancer effects of free polyunsaturated fatty acids in an oily lymphographic agent following intrahepatic arterial administration to a rabbit bearing VX-2 tumor.. Cancer Res.

[OCR_00727] Hennessy C., Henry J. A., May F. E., Westley B. R., Angus B., Lennard T. W. (1991). Expression of the antimetastatic gene nm23 in human breast cancer: an association with good prognosis.. J Natl Cancer Inst.

[OCR_00732] Holm R., Høie J., Kaalhus O., Nesland J. M. (1995). Immunohistochemical detection of nm23/NDP kinase and cathepsin D in medullary carcinomas of the thyroid gland.. Virchows Arch.

[OCR_00737] Honn K. V., Nelson K. K., Renaud C., Bazaz R., Diglio C. A., Timar J. (1992). Fatty acid modulation of tumor cell adhesion to microvessel endothelium and experimental metastasis.. Prostaglandins.

[OCR_00742] Honn K. V., Tang D. G., Gao X., Butovich I. A., Liu B., Timar J., Hagmann W. (1994). 12-lipoxygenases and 12(S)-HETE: role in cancer metastasis.. Cancer Metastasis Rev.

[OCR_00750] Howlett A. R., Petersen O. W., Steeg P. S., Bissell M. J. (1994). A novel function for the nm23-H1 gene: overexpression in human breast carcinoma cells leads to the formation of basement membrane and growth arrest.. J Natl Cancer Inst.

[OCR_00756] Hsu S., Huang F., Ossowski L., Friedman E. (1995). Colon carcinoma cells with inactive nm23 show increased motility and response to motility factors.. Carcinogenesis.

[OCR_00761] Iizuka N., Oka M., Noma T., Nakazawa A., Hirose K., Suzuki T. (1995). NM23-H1 and NM23-H2 messenger RNA abundance in human hepatocellular carcinoma.. Cancer Res.

[OCR_00781] Jiang W. G., Bryce R. P., Mansel R. E. (1997). Gamma linolenic acid regulates gap junction communication in endothelial cells and their interaction with tumour cells.. Prostaglandins Leukot Essent Fatty Acids.

[OCR_00766] Jiang W. G., Hiscox S., Hallett M. B., Scott C., Horrobin D. F., Puntis M. C. (1995). Inhibition of hepatocyte growth factor-induced motility and in vitro invasion of human colon cancer cells by gamma-linolenic acid.. Br J Cancer.

[OCR_00793] Kantor J. D., McCormick B., Steeg P. S., Zetter B. R. (1993). Inhibition of cell motility after nm23 transfection of human and murine tumor cells.. Cancer Res.

[OCR_00803] Leone A., Flatow U., King C. R., Sandeen M. A., Margulies I. M., Liotta L. A., Steeg P. S. (1991). Reduced tumor incidence, metastatic potential, and cytokine responsiveness of nm23-transfected melanoma cells.. Cell.

[OCR_00808] Leone A., Flatow U., VanHoutte K., Steeg P. S. (1993). Transfection of human nm23-H1 into the human MDA-MB-435 breast carcinoma cell line: effects on tumor metastatic potential, colonization and enzymatic activity.. Oncogene.

[OCR_00819] MacDonald N. J., De la Rosa A., Benedict M. A., Freije J. M., Krutsch H., Steeg P. S. (1993). A serine phosphorylation of Nm23, and not its nucleoside diphosphate kinase activity, correlates with suppression of tumor metastatic potential.. J Biol Chem.

[OCR_00826] MacDonald N. J., de la Rosa A., Steeg P. S. (1995). The potential roles of nm23 in cancer metastasis and cellular differentiation.. Eur J Cancer.

[OCR_00831] Maehle L., Eilertsen E., Mollerup S., Schønberg S., Krokan H. E., Haugen A. (1995). Effects of n-3 fatty acids during neoplastic progression and comparison of in vitro and in vivo sensitivity of two human tumour cell lines.. Br J Cancer.

[OCR_00837] Mandai M., Konishi I., Komatsu T., Mori T., Arao S., Nomura H., Kanda Y., Hiai H., Fukumoto M. (1995). Mutation of the nm23 gene, loss of heterozygosity at the nm23 locus and K-ras mutation in ovarian carcinoma: correlation with tumour progression and nm23 gene expression.. Br J Cancer.

[OCR_00843] Martinez J. A., Prevot S., Nordlinger B., Nguyen T. M., Lacarriere Y., Munier A., Lascu I., Vaillant J. C., Capeau J., Lacombe M. L. (1995). Overexpression of nm23-H1 and nm23-H2 genes in colorectal carcinomas and loss of nm23-H1 expression in advanced tumour stages.. Gut.

[OCR_00815] McIllmurray M. B., Turkie W. (1987). Controlled trial of gamma linolenic acid in Duke's C colorectal cancer.. Br Med J (Clin Res Ed).

[OCR_00849] Newman M. J. (1990). Inhibition of carcinoma and melanoma cell growth by type 1 transforming growth factor beta is dependent on the presence of polyunsaturated fatty acids.. Proc Natl Acad Sci U S A.

[OCR_00861] Parhar R. S., Shi Y., Zou M., Farid N. R., Ernst P., al-Sedairy S. T. (1995). Effects of cytokine-mediated modulation of nm23 expression on the invasion and metastatic behavior of B16F10 melanoma cells.. Int J Cancer.

[OCR_00866] Parish C. R., Jakobsen K. B., Coombe D. R. (1992). A basement-membrane permeability assay which correlates with the metastatic potential of tumour cells.. Int J Cancer.

[OCR_00885] Rose D. P., Connolly J. M., Meschter C. L. (1991). Effect of dietary fat on human breast cancer growth and lung metastasis in nude mice.. J Natl Cancer Inst.

[OCR_00890] Rosengard A. M., Krutzsch H. C., Shearn A., Biggs J. R., Barker E., Margulies I. M., King C. R., Liotta L. A., Steeg P. S. (1989). Reduced Nm23/Awd protein in tumour metastasis and aberrant Drosophila development.. Nature.

[OCR_00895] Royds J. A., Cross S. S., Silcocks P. B., Scholefield J. H., Rees R. C., Stephenson T. J. (1994). Nm23 'anti-metastatic' gene product expression in colorectal carcinoma.. J Pathol.

[OCR_00900] Schneider M. R., Tang D. G., Schirner M., Honn K. V. (1994). Prostacyclin and its analogues: antimetastatic effects and mechanisms of action.. Cancer Metastasis Rev.

[OCR_00905] Simpson J. F., O'Malley F., Dupont W. D., Page D. L. (1994). Heterogeneous expression of nm23 gene product in noninvasive breast carcinoma.. Cancer.

[OCR_00923] Steeg P. S., de la Rosa A., Flatow U., MacDonald N. J., Benedict M., Leone A. (1993). Nm23 and breast cancer metastasis.. Breast Cancer Res Treat.

[OCR_00926] Takeda S., Horrobin D. F., Manku M., Sim P. G., Ells G., Simmons V. (1992). Lipid peroxidation in human breast cancer cells in response to gamma-linolenic acid and iron.. Anticancer Res.

[OCR_00931] Takeda S., Sim P. G., Horrobin D. F., Sanford T., Chisholm K. A., Simmons V. (1993). Mechanism of lipid peroxidation in cancer cells in response to gamma-linolenic acid (GLA) analyzed by GC-MS(I): Conjugated dienes with peroxyl (or hydroperoxyl) groups and cell-killing effects.. Anticancer Res.

[OCR_00939] Tiwari R. K., Mukhopadhyay B., Telang N. T., Osborne M. P. (1991). Modulation of gene expression by selected fatty acids in human breast cancer cells.. Anticancer Res.

[OCR_00944] Tokunaga Y., Urano T., Furukawa K., Kondo H., Kanematsu T., Shiku H. (1993). Reduced expression of nm23-H1, but not of nm23-H2, is concordant with the frequency of lymph-node metastasis of human breast cancer.. Int J Cancer.

[OCR_00950] Van der Merwe C. F., Booyens J., Katzeff I. E. (1987). Oral gamma-linolenic acid in 21 patients with untreatable malignancy. An ongoing pilot open clinical trial.. Br J Clin Pract.

[OCR_00961] Venturelli D., Martinez R., Melotti P., Casella I., Peschle C., Cucco C., Spampinato G., Darzynkiewicz Z., Calabretta B. (1995). Overexpression of DR-nm23, a protein encoded by a member of the nm23 gene family, inhibits granulocyte differentiation and induces apoptosis in 32Dc13 myeloid cells.. Proc Natl Acad Sci U S A.

[OCR_00969] Viel A., Dall'Agnese L., Canzonieri V., Sopracordevole F., Capozzi E., Carbone A., Visentin M. C., Boiocchi M. (1995). Suppressive role of the metastasis-related nm23-H1 gene in human ovarian carcinomas: association of high messenger RNA expression with lack of lymph node metastasis.. Cancer Res.

[OCR_00976] Wang L., Patel U., Ghosh L., Chen H. C., Banerjee S. (1993). Mutation in the nm23 gene is associated with metastasis in colorectal cancer.. Cancer Res.

[OCR_00981] Xerri L., Grob J. J., Battyani Z., Gouvernet J., Hassoun J., Bonerandi J. J. (1994). NM23 expression in metastasis of malignant melanoma is a predictive prognostic parameter correlated with survival.. Br J Cancer.

[OCR_00986] Yamaguchi A., Urano T., Fushida S., Furukawa K., Nishimura G., Yonemura Y., Miyazaki I., Nakagawara G., Shiku H. (1993). Inverse association of nm23-H1 expression by colorectal cancer with liver metastasis.. Br J Cancer.

[OCR_00880] de la Rosa A., Williams R. L., Steeg P. S. (1995). Nm23/nucleoside diphosphate kinase: toward a structural and biochemical understanding of its biological functions.. Bioessays.

[OCR_00954] van der Merwe C. F., Booyens J., Joubert H. F., van der Merwe C. A. (1990). The effect of gamma-linolenic acid, an in vitro cytostatic substance contained in evening primrose oil, on primary liver cancer. A double-blind placebo controlled trial.. Prostaglandins Leukot Essent Fatty Acids.

